# Health care professionals’ perspectives on screening and management of gestational diabetes mellitus in public hospitals of South India – a qualitative study

**DOI:** 10.1186/s12913-021-06077-0

**Published:** 2021-02-12

**Authors:** Biswamitra Sahu, Giridhara R. Babu, Kaveri Siddappa Gurav, Maithili Karthik, Deepa Ravi, Eunice Lobo, Daisy Abu John, Laura Oakley, Eugene Oteng-Ntim, Iliatha Papachristou Nadal, Sanjay Kinra

**Affiliations:** 1 Indian Institute of Public Health-Bengaluru, Public Health Foundation of India (PHFI), Magadi Rd 1st cross, Next to leprosy hospital, SIHFW premises, Bengaluru, Karnataka 560023 India; 2 Wellcome Trust-DBT India Alliance Intermediate Research Fellow in Public Health, Bengaluru, India; 3 London School of Hygiene and Tropical Medicine, London, UK; 4 Centre for Fertility and Health, Norwegian Institute of Public Health, Oslo, Norway; 5 Department of Women’s Health, Guy’s and St Thomas’ NHS Foundation Trust (King’s Health Partners), St Thomas’ Hospital, Westminster Bridge Road, London, UK

**Keywords:** Gestational diabetes mellitus, Screening, Management, Health care providers, India, Public sector, Socio-ecological model

## Abstract

**Background:**

Women developing Gestational Diabetes Mellitus (GDM) are subsequently at a higher risk of developing Type 2 Diabetes later in life. Screening and effective management of women with GDM are essential in preventing progression to type 2 diabetes mellitus. We aimed to explore the perspectives of healthcare providers regarding the barriers from the health system context that restrict the timely screening and effective management of GDM.

**Methods:**

We conducted six in-depth interviews of health care providers- four with nurses and two with obstetricians in the public hospitals in India’s major city (Bengaluru). The interviews were conducted in the preferred language of the participants (*Kannada* for nurses, English for the obstetricians) and audio-recorded. All *Kannada* interviews were transcribed and translated into English for analysis. The primary data were analyzed using the grounded theory approach by NVivo 12 plus. The findings are put into perspective using the socio-ecological model.

**Results:**

Health care providers identified delayed visits to public hospitals and stress on household-level responsibilities as barriers at the individual level for GDM screening. Also, migration of pregnant women to their natal homes during first pregnancy is a cultural barrier in addition to health system barriers such as unmet nurse training needs, long waiting hours, uneven power dynamics, lack of follow-up, resource scarcity, and lack of supportive oversight. The barriers for GDM management included non-reporting women to follow - up visits, irregular self-monitoring of drug and blood sugar, trained staff shortage, ineffective tracking, and lack of standardized protocol.

**Conclusion:**

There is a pressing need to develop and improve existing GDM Screening and Management services to tackle the growing burden of GDM in public hospitals of India.

**Supplementary Information:**

The online version contains supplementary material available at 10.1186/s12913-021-06077-0.

## Background

Gestational Diabetes Mellitus (GDM) is the presence of higher blood glucose levels diagnosed for the first time during pregnancy. Globally, GDM affects 18.4 million live births, with 4 million in India [1, 2]. Women with GDM are at a higher risk of adverse health consequences in pregnancy and beyond [3]. Children born of women with GDM have a higher risk of obesity and type 2 diabetes mellitus (T2DM) [4]. The International Association of the Diabetes and Pregnancy Study Groups (IADSPG) recommends conducting an Oral Glucose Tolerance Test (OGTT) during 24–28 weeks of pregnancy for the screening of GDM [5, 6]. In India, there is limited evidence regarding the coverage and quality of GDM screening and management (GDMSM). As a result, the reported prevalence of GDM varies between null to 41.9% [7].

Timely screening and adequate treatment of GDM can minimize the impact on adverse consequences. When GDM is diagnosed early, evidence-based lifestyle interventions can reduce the risk of developing Type 2 Diabetes Mellitus(T2DM) in both the mother and baby [8]. The lack of uniformity in GDMSM services in India is striking. Earlier, we reported that there is poor compliance in following the GDMSM recommendations by health care providers in India [9]. We demonstrated that the prevalence of GDM in public hospitals according to current screening practices was merely 4.3%, compared to a prevalence of 15.4% when universal screening was undertaken [10]. Also, only one in five women diagnosed with GDM receives some follow-up treatment in public hospitals [10]. In a previous paper, we reported that public hospitals missed 70% of women from GDM diagnosis. The high prevalence of GDM in India is driven by a huge prevalence of obesity and being overweight in India [11]. Nearly, 4.3 million pregnant women are estimated to be overweight or obese, and the prevalence of obesity among women has risen from 16.6% in 2005 to 21.7% in 2014 [12, 13]. It is expected that the GDM prevalence in India will continue to rise in the future. Therefore, there is an urgent need to understand and implement GDM screening and management services in Indian public hospitals.

Public hospitals in India provide healthcare services to nearly 40% of the population [14]. In India, the secondary level health care centers include District Hospitals and Community Health Centre (CHC) at the block level. Tertiary Health care refers to specialized consultative care, ideally provided when referrals are made from primary and secondary hospitals. However, most tertiary hospitals also have to provide primary and secondary level health care in addition to specialty health services. An obstetrician at a tertiary hospital has to examine nearly 30 to 50 pregnant women during a typical workday. In many public hospitals, there are no obstetricians, and general practitioners do antenatal examinations. There is also a severe shortage of workforce; 74.8% CHCs are functioning without an obstetrician in India [15], thus resulting in suboptimal quality of antenatal services. Secondary and tertiary level public hospitals in urban areas are overcrowded and often create physician burnout. A qualitative study reported the perceived poor quality of health care facilities as a detrimental factor influencing women’s healthcare-seeking behavior [16].

Health care providers (HCPs) are subjected to stress due to heavy workloads from over-crowding in public hospitals. Screening for GDM during pregnancy was not found to be universal in two states of India, namely, Haryana and Andhra Pradesh [17]. This study revealed deviation from protocol both by pregnant women as well as HCPs. For instance, pregnant women did not report for screening with an empty stomach which forced HCPs to deviate from the screening guideline and opting for testing random blood sugar (RBS). If the RBS value was above 160 mg/dl – an arbitrary cut-off decided by HCPs for referral of the case to a physician. Also, rural areas of the study reported a lack of facilities for conducting the OGTT test. A study among clinicians in the state of Kerala highlights that the HCPs were not following the national guidelines while managing the GDM cases. HCPs observed that due to family responsibilities women with GDM were not able to come for follow-up care [18, 19]. The socio-ecological model is a generic framework for exploring the interconnection between different levels in explaining a problem at hand. The socio-ecological model has not been developed for understanding barriers to timely screening and management of GDM. However, it is useful for understanding dietary patterns among those living with diabetes [20].

Such research is valuable, clarifying the challenges they face in delivering healthcare, and ascertaining their insights on how barriers can be addressed. The use of qualitative methods can reveal the perspectives from the experiences of those who are agents of change in GDMSM services. Also, this insight drawn from qualitative methodology can be immensely useful for incorporating a participatory dimension for the development of health interventions. It can provide context-driven information to guide and inform the development of services for GDMSM. However, there is limited evidence regarding the perspectives of HCPs in ensuring effective GDMSM services in public hospitals. Currently, the contextual nature of the clinical practices relating to the screening and management of women with GDM in India is poorly understood. We aimed to understand the context and experiences of health system stakeholders in GDMSM services. The results of this study will contribute to the design and implementation of a feasible and sustainable educational intervention for women with GDM within the context of public hospitals in India.

## Methods

### Study setting

Data were collected between March to June 2019 from two selected public hospitals in Bengaluru, South India. These two hospitals cater mostly to women from lower socio-economic backgrounds. Jayanagar General Hospital (JGH) is a 300-bedded tertiary care facility with eight obstetricians, with nearly 500 delivery monthly and provision of a Neonatal Intensive Care Unit (NICU). Srirampura Referral Hospital (SRRH) is a 30-bedded CHC, located adjacent to a neighboring slum. The hospital has three obstetricians, with most high-risk pregnancy cases, particularly GDM cases being referred to tertiary centers for the subsequent management and delivery. Both the facilities reportedly follow national guidelines in screening for and managing GDM. These hospitals also endorse the ongoing cohort study, entitled “Maternal Antecedents of Adiposity and Studying the Transgenerational role of Hyperglycemia and Insulin” (MAASTHI) [21].

### Participants

The study participants comprised of two obstetricians and four nurses, identified from the 2 hospitals of MAASTHI project [21]. The six participants were willing to participate and met our participant selection criteria, namely, HCPs with five or more years of work experience, and who had experience (at least 1 year) of providing GDM services. The sample was drawn purposively as the number of experienced obstetricians is limited and they keep busy at government health facilities. However, despite their busy schedule, we had two Obstetricians and four nurses willing to participate from both locations. We stopped recruiting after interviewing six participants because they met our study criteria and we obtained saturation in terms of the depth of information that we were seeking to unravel. All of them were experienced in delivering care to pregnant women with GDM in public hospitals. The details of these 6 participants are provided in Table 1.

**Table 1:** Profile of health care providers interviewed

Sl No	Age	Designation	Education	Experience (in years)
1	30–40	Obstetrician	MBBS, OBG	5–10
2	40–50	Staff nurse OBG	BSc Nursing	5–10
3	60–70	Obstetrician	MBBS, DGO, MD, PGDMLE, MICOG	40–50
4	50–60	Senior staff Nurse OBG	BSc Nursing	20–30
5	50–60	Senior Staff Nurse	BSc Nursing	20–30
6	50–60	Senior staff nurse	BSc Nursing	20–30

All the nurses and obstetricians approached provided written informed consent to participate in the interviews. After permission, all interviews were audio-recorded, and detailed notes were taken throughout the discussions.

### Interview instruments

The topic guides for the interviews were developed through a pilot study for a larger trial assessing a film-based intervention for improving screening and management of GDM. The topic guides were designed to explore knowledge, skills, and resources concerning GDM screening and management, with domains on GDM knowledge, perception about GDM patients, GDM screening process, and the HCPs’ role in GDM management, and available resources.

### Data collection

Experienced and trained researchers conducted data collection. The lead researcher, an experienced qualitative researcher trained team members on the study protocol, instrument, and interview techniques. The two interviewers are women and well conversant in English and Kannada - the local language of the study site. They both are well trained in qualitative methods with rich experience of using the method in data collection, processing, and analyzing. They have worked on varied topics around the theme of woman’s health and have an inherent interest in the topic of this study.

Each interview lasted for approximately 30 to 80 min. All the interviews for the obstetricians were conducted in English; for nurses, the preferred local language (*Kannada*) was used.

### Data processing and analysis

Interviews continued until data saturation was obtained. Nurse interviews were transcribed into *Kannada* and then translated into English by researchers, and analysis was performed using the English transcripts. The data were analyzed using grounded theory. Traditionally, the Grounded theory employed an inductive procedure of data analysis [22]; however, we have used the analytical cycle [23] that consists of both inductive and deductive techniques of theory development. We have coded transcripts, labelled and categorized concepts, connected categories and subcategories, and integrated the prime categories to develop a coherent narrative emerging from the empirical data. The Socio-ecological model has formed the basis of the in-depth interview guide. The questions in the guide were developed at different levels. These questions formed a priori codes and aided the development of themes during the analysis. The Socio-ecological model was used to connect themes and develop the induced theoretical framework. We have used the Socio-ecological model to understand the interrelationship between individual, household, cultural and health system barriers that inhibit timely screening of GDM and its management. The data were analyzed using NVivo (Version 12 plus).

## Results

Analysis of the interviews with HCPs revealed their perceptions regarding GDM screening; their understanding of current GDM screening guidelines and to what extent these were being followed; their knowledge of GDM and the source of this knowledge, and their recommendations on how GDM screening practices can be improved.

### Perceived importance of screening for GDM

All HCPs interviewed emphasized the importance of screening pregnant women for GDM. They laid stress on early detection of GDM, commenting that prompt detection could minimize possible complications during delivery. One of the obstetricians mentioned that GDM screening was introduced a few years ago in the hospital and had noticeably reduced the risks associated with delivery.

*“It is very good that we are diagnosing it (GDM) very early, the risk has been reduced so, it is very important; most of the maternal risk have been reduced. Since we diagnose early, we advise her (pregnant woman) diet and exercise early, and even we start metformin in an early stage” (IDI#3, Obstetrician)*

A nurse participant elaborated on the potential advantages of knowing GDM status through screening as follows:

*“It is easy if known (GDM status), it is easy to manage such cases. We can give more preference to such cases. If she is in line with many other women who are due for delivery, we can give preference to the one with GDM and prepare for the labor, and take necessary precautions. Like we can give her the needed medicines, inform pediatricians to attend to the baby soon after the delivery” (IDI#4, Nurse).*

### Current practices aligned with national guidelines

The obstetricians strongly supported the national recommendation of staged screening, in which women in the first trimester are given the Glucose Challenge Test (GCT), and those testing negative are screened again using a Glucose Tolerance Test (GTT) between 24th and 28th week. Furthermore, women who test negative using GTT are additionally asked to undergo Fasting Blood Sugar (FBS) and Post Prandial Blood Sugar (PPBS) tests at 32 weeks. A comparative analysis of the National Health Mission in India, WHO guidelines, and FIGO guidelines is presented in Table 2. The women who come late to the hospital during pregnancy (in the second trimester) are recommended for GTT.

**Table 2:** Comparative guidelines for GDM management, National Health Mission in India, WHO and FIGO

Name	Diagnosis	Management
**Guideline prescribed by the National Health Mission in India**	Demand generation – Community awareness, Sensitization for GDM, and client mobilization. Diagnosis – The first GDM testing OGTT at first ANC contact and if < 140 mg/ dL, then second testing OGTT at 24–28 weeks of pregnancy.	Management – If the OGTT result is ≥140 mg/dL then start MNT and exercise on the same day. Start medical management if PPBS result ≥120 mg/dL in a subsequent follow-up visit. Follow-up – PPBS monthly till delivery. Ultrasonography at 18–20, 28–30 & 34–36 weeks of pregnancy. Referral – As per the reasons cited in the guideline [24]
**WHO guidelines**	Diabetes mellitus in pregnancy should be diagnosed by the 2006 WHO criteria for diabetes if one or more of the following criteria are met: • fasting plasma glucose ≥7.0 mmol/l (126 mg/ dl) • 2-plasma glucose ≥11.1 mmol/l (200 mg/dl) following a 75 g oral glucose load • random plasma glucose ≥11.1 mmol/l (200 mg/ dl) in the presence of diabetes symptoms. The diagnosis of gestational diabetes mellitus at any time during pregnancy should be based on any one of the following values: • Fasting plasma glucose = 5.1–6.9 mmol/l (92–125 mg/dl) • 1-h post 75 g oral glucose load > = 10.0 mmol/l (180 mg/dl)* • 2-h post 75 g oral glucose load 8.5–11.0 mmol/l (153–199 mg/dl)	Medical Nutrition Therapy and Insulin Therapy/Metformin as required [25]
**FIGO guidelines**	All women at booking/first trimester-Measure FPG, RBG, or HBA1c to detect diabetes in pregnancy In 24–28 weeks, if it turns to be negative, perform 75-g 2-h OGTT	-If lifestyle modification fails, metformin, glyburide, or insulin should be considered as safe and effective treatment options for GDM -Self-monitoring of blood glucose is recommended for all pregnant women with diabetes, 3–4 times a day: Fasting: Once daily, following at least 8 h of overnight fasting. Postprandial: 2–3 times daily, 1 or 2 h after the onset of meals, rotating meals on different days of the week Self-monitoring of the blood glucose is recommended for all pregnant women with diabetes at least once daily, with documented relation to the timing of the meal. Recommendations for insulin treatment in women with gestational diabetes mellitus: -The following insulin may be considered safe and effective treatment during pregnancy: Regular insulin, NPH, lispro, aspart, and detemir [26]

*“We have our setup- free of cost. For GCT, before it was done in a fasting state, but now they (women) can come at any time. Even she can take a test irrespective of fasting status”[sic] (IDI#3, Obstetrician)*

Obstetricians did not refute the World Health Organisation (WHO) guidelines that recommend women undergo OGTT on an empty stomach (fasting state) [27]; however, they felt that GCT is better suited for pregnant Indian women in the first trimester. According to them, the test is ‘simple’ and ‘practical’, as it does not require pregnant women to visit the hospital to take the test on an empty stomach. The participating obstetricians recommend the OGTT (available under the MAASTHI project) for pregnant women who visit the hospital in and after the second trimester.

The nurses’ knowledge of GDM guidelines were limited. Their role in GDM screening or management is not well defined, and they have clear compartmentalization of tasks where the obstetrician prescribes and they follow instructions. This was expressed by a nurse as follows:

*“After the test, women go to obstetricians. Obstetricians provide prescriptions and dietary advice. They provide treatment and tell them to come for follow-ups. We do not advise women, as this is more the role of the obstetrician. Nor does the patient ask us (nurse). If one patient is diagnosed with GDM, we register and send them to madam (Obstetrician). They advise. If patients do not understand what is written, only then do we read prescriptions and tell them what to do, such as how to take medicine.” (IDI#2, Nurse).*

### Nature of GDM knowledge

The obstetricians were well-informed regarding the causes, consequences, and management of GDM. Additionally, they were also aware of national guidelines and the protocols for screening and GDM management.

*“There are two types of women, suppose if they (women) come in the first trimester we follow the Government of India guidelines, we give them 75 grams of oral glucose and check for glucose after two hours. This test is very suitable for the Indian population. If they miss and come in the second trimester, then we follow WHO guidelines, then we refer to MAASTHI cohort to do the test. They are doing research and they will counsel and then they will tell them to come on a particular day on empty stomach” (IDI#3, Obstetrician).*

The obstetricians reported that they keep themselves abreast of the latest developments; specifically, national guidelines are regularly updated through Continued Medical Education programs. They did not feel that any further training on GDM screening and management was required. However, the nurses reported that relevant training was lacking, and they expressed interest in receiving formal training on GDM specifically designed for the nurse role.

### Recommended methods to educate women about GDM

All HCPs admitted that they have very little time to educate pregnant women and their family members regarding GDM. However, they suggested means through which additional GDM related information could be disseminated. This included mass media (newspapers and Television), display of posters in hospital premises, telecasting educational films in the hospital, and dedicated counselors to educate about GDM. However, the most highly recommended option was showing educational films about GDM in the hospital on Antenatal care (ANC) days. The HCPs were keen on this approach as such films could be designed to be accessible and easily understood, and women would be able to watch it during their waiting time at the clinics. Additionally, it was felt that messages provided through the film on hospital premises would be taken seriously.

**Fig. 1:**
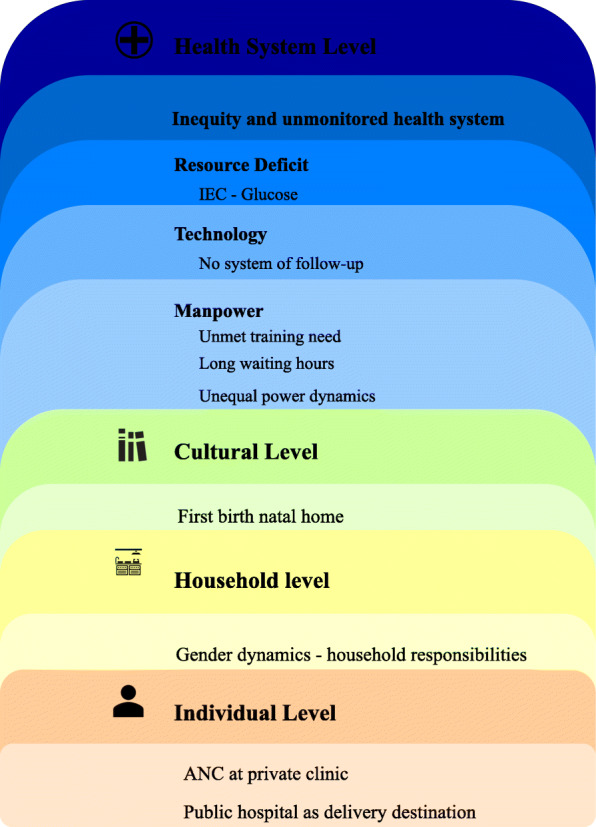
Socio-ecological model explaining the barriers to screening of GDM in public hospitals of Bengaluru

The HCPs have reiterated that screening of GDM is not happening on time and that has the potential to adversely impact the management of GDM. In the section below we delve deep into the barriers that impede timely screening of GDM (Fig. 1).

### Barriers to the timely screening of GDM

The findings of our study that explore the barriers to a timely screening of GDM from an HCP’s perspective are organized around different levels of the socio-ecological model, namely, at the individual, household gender dynamics, culture, and health system levels. A barrier to the timely screening of GDM at the individual level is that pregnant women access ANC at private clinics due to convenience. Unfortunately, these small clinics often fail to screen for GDM. Further, pregnant women choose to report at the public hospital, where GDM screening is available, in late gestation as a delivery destination and miss out on a timely screening of GDM. The household gender dynamics contributes towards the individual level barrier, for instance, women deliver parity two and above in their marital home where they have household responsibilities, due to which they choose health facility closer home in early gestation as it allows them to save time which they might have otherwise spent on traveling to a health facility or braving long waiting time. However, they choose a tertiary level public hospital for the delivery because that is better equipped for ensuring favorable birth outcomes. At a cultural level, these pregnant women migrate to deliver their firstborn at their natal home due to local practices. However, since they migrate when they are approaching the expected date of delivery, they report late for GDM screening at the tertiary level public hospital situated at their natal home. Hence, gets delayed for the screening of GDM. At the health system level, the barriers to the timely screening of GDM can be sub-categorized as human resources, technology, resource deficit, and unmonitored health system. The human resource can further be classified as an unmet training need of nurses in GDM and long waiting hours. Due to a shortage of health staff, there is overcrowding at the health facility and the prime reason for long waiting time; thus, is a deterrent for the timely screening of GDM. Again, the existing health system lacks the technology that is required to follow up deferred cases of pregnant women who have missed the screening of GDM due to nausea or some other health issue. The resource deficit, such as the unavailability of GDM health promotion material further fails to generate demand for GDM screening among pregnant women. Finally, there exists widespread inequity in access to GDM screening and management facilities specifically for women located in rural locations because of the unavailability of such services. Also, unmonitored medical pluralism creates confusion for many pregnant women in accessing the right information and guidance they need for ensuring optimal management of GDM. A more detailed thematic elaboration of the socio-ecological model that details the barrier to a timely screening of GDM from the HCP’s perspective that has emerged from the analysis is presented in the following section.

### Individual-level barrier

The individual-level barriers are availing ANC at the private clinic and public hospital as a delivery destination.

#### Availing ANC at the private clinic in the first trimester

HCPs described how women visiting the public hospital in their second trimester or later have usually availed ANC at private clinics in the first trimester and such private facilities have not guided them to undergo GDM screening. A nurse threw some light on this as follows:

*“When people go to small clinics where there are no obstetricians, obstetricians in such clinics usually do not write (prescribe) for blood tests, rather think that scanning is important. They (women) would have undergone 7 to 8 scans. I can give you a proof of this. Sometimes I scold them and ask why you have done so many (scans)? And why have you not done single blood tests, for that they say that the doctor had not told them” (IDI #1, Nurse)*

As a result, many of these women miss out on GDM screening at the early stages of pregnancy. The nurse participant has also touched upon a pertinent ethical violation that is occurring in the private medical sector where due to the economic reason these pregnant women are advised to undergo several scans which might not be medically indicated. However, in the current scheme of things, the screening of GDM gets neglected as highlighted by the participant. If the screening is not done on time, this will be the most significant impediment for effective management of GDM, as these women are not aware of their condition.

#### Public hospitals are seen as a delivery destination only

Some pregnant women who have already had the experience of childbirth through their earlier pregnancies visit the public hospitals considering it as a delivery destination only; therefore have delayed seeking services from the tertiary and community health facilities and thus miss out on timely GDM screening.

*“( … … ) Later (four to five days before the due date) for the delivery they come to the government hospital. For such cases, I pay more attention to see if they have even undergone any blood tests or sugar test (because chances of them not having blood test reports are higher.” (IDI#4, Nurse).*

Antenatal mothers visit private clinics for initial ANC (after 6 to 7 months), and when they are near the date of delivery, they visit the public hospital with plans to deliver. At this advanced stage of pregnancy detection of GDM is delayed.

#### Household-level gender dynamics barrier

The gender dynamics at the household level places many impediments before these pregnant women in addressing their health concerns. They are busy attending to the needs of others and often undermine their well-being. Consequently, they prioritize household responsibilities hence fall short of time and find it convenient to visit a health facility that is closer home. According to a nurse participant, women are only getting screened for GDM between 6 and 8 months of pregnancy.

*“Women do not go to mother’s place for the second or third delivery, (stay with the husband’s family, where they have many responsibilities to fulfill). So, in that situation, women usually go to the clinic nearby (to their husband’s place) for a routine check-up (up to 9*^*th*^ *month) … … … ” (IDI#4, Nurse).*

Visiting a nearby clinic for ANC ensures better time management for addressing family responsibilities; however, at these health facilities, they are less likely to be guided towards getting screened for GDM at the first place and also in at the right time.

### Culture level barrier

Culture plays a crucial role in dictating the norms around pregnancy, delivery, and postnatal care. There are several dos and don’ts outlined through oral tradition that people follow revolving around a pregnant woman’s nutrition, ritual, rites of passage, work, movement, social interaction, and emotional support to name a few. The first birth has a prominent cultural significance.

#### The first birth at natal home

It is a cultural practice in Karnataka that women are sent to their maternal home for delivery of their firstborn child. In our study, women travel from their marital homes to deliver in Bengaluru, usually at 6 months of pregnancy or later. These pregnant women then report late in pregnancy at the government hospital near their maternal home. Since they have not undergone screening of GDM at the clinics near their marital home, they are late for the screening of GDM at these tertiary and Community Health Centres.

### Health system-level barrier

At the health system level, the barriers to a timely screening of GDM can be sub-categorized as human resources, technology, resource deficit, and unmonitored health system.

### Human resource

Adequate and trained human resource is the backbone of any well-functioning health system. When the basics of the health system are not in place then our aspiration for the timely screening of GDM will face a roadblock. The three main human resource issues that were identified at the research location are the a) unmet training need of nurses in GDM b) long waiting hours due to shortage of paramedic staff c) unequal power dynamics.

#### Unmet training need of nurses in GDM health promotion

A nurse participant at the tertiary care felt confident that she had all the necessary practical information regarding the appropriate management of GDM and how to prepare to care for a woman with GDM during delivery and post-delivery:

*“I tell women to get the blood test after one week of taking medicine/ insulin, so that they know if sugar levels decreased/ increased, ( … … … … … … … ) we tell them to control sugar levels through diet control and by taking medicine correctly ( … … .). And then, for those women who come for delivery, we check if they have taken insulin and how much had they taken. ( … … .)*

*After delivery, we recommend women to take General Random Blood Sugar (GRBS) test 2 times. Suppose a woman’s sugar level is not under control, we put them on insulin. Those women, who deliver through C-section, usually stay for 5 days in the hospital, so we check and monitor their sugar level every day. And then after 40 days, we ask them to come to the hospital for a check-up.” (IDI#5, Nurse).*

However, all the nurses stressed the importance of obtaining formal training on how to educate and support pregnant women, emphasizing that such training would ensure they share the correct information. A nurse participant elaborates the need for training as follows:

*“It would be very helpful if we get some training on GDM treatment, we (nurse) will be in OPD (Out Patient Department), and there we see many pregnant women. ( … … ..). Now when they ask, we are providing some information, but we need the training to provide correct information, and we also need training on how to give correct information. If you organize any class (training) we will attend that”. (IDI#5, Nurse).*

Another nurse participant stresses the need for training and emphasizes how this training can help advert adverse consequences of GDM as follows:

*“We haven’t had any training but we need it. We have more GDM cases but have very little information. It will be useful. We can also provide information in our neighborhood. People will be interested to know ( … … .). We can reduce death, improve mother and child health, and conduct the delivery nicely”. (IDI#1, Nurse).*

#### Long waiting hours

Another important challenge for effective screening and management of GDM is the long waiting hours that pregnant women experience while availing of service at public hospitals. For instance, a nurse elaborates how women forget to collect their blood test report and also recognized the long waiting hours being a reason for that as follows:

*“We tell women to get blood tested, she says yes, but she goes home without being tested.*

*It is negligence, whether educated or not educated, they do not like to wait. ( … … … ..)Laboratory people go for lunch at 1 pm, and they do not conduct any tests after they come back from lunch. So, when they (women) come for their next ANC, when we ask for lab reports, they say “I have not undergone test yet and I will get it today. Then I send them back for the blood test and only after that I send them for ANC check-up. This delays early detection sometimes.” (IDI#1, Nurse)*

This is an inadequacy at the health system level in that there are long waiting hours that pregnant women have to endure to collect the result of their GDM screening. It may be due to a lack of planning at the health system level where seamless service is not ensured in dispensing GDM screening reports. However, the nurse participant firstly blames the pregnant women for being negligent as they fail to collect their report on time. We already know from the household level gender dynamics the challenges that women face in balancing their household responsibilities and paying adequate attention to their health. Hence, when they experience a time crunch, it is likely that they forgo waiting as they have pressing issues to be attended at home.

*“One thing is that they (women) have to wait in a long line. And they have to wait for 3 hours to get the report. So, they usually do not collect on the day they get tested Instead they go off thinking that they can always collect it next time. Then they forget and come directly for a check-up, some of them go to their mothers/ husband’s place, and the report will be lying here for months. Sometimes a woman would have gotten tested in another place and then she comes here with other reports but not the diabetes report. These things happen we need to find a solution to this.”(IDI#1, Nurse)*

Apart from long waiting hours, there could be a problem at the individual level where pregnant women do not collect reports on time either due to ignorance regarding the importance of test, negligence, or cultural level reasons where they migrate to their natal home for delivery. The nurse participant has a reflective tone in the above verbatim where she stresses the need to address this challenge at the health system level.

#### Unequal power dynamics

Unequal power dynamics between the health care provider and those seeking care have the potential to jeopardize the quality of care. The tone and manner of speech used by health care providers can act as a deterrent for those seeking care as it has the potential to interfere in establishing respect and trust for the information shared by the health staff. Also, the verbatim below bares open the power dynamics that could exist between health care providers and those seeking health care where she is sarcastic that pregnant women come to government hospitals and are not prepared to wait.

*They will not have time (to collect blood reports), they come to government hospitals and they don’t have time! They cannot wait in line in front of the laboratory. ”(IDI#1, Nurse)*

A health system challenge is expressed in a matter of fact manner without any attempt to own up the situation because of this unequal power dynamics between provider and recipient of health service. This could be a potential barrier in disseminating GDM related health promotion messages or directly helping them through the consultation procedure.

### Technology

Technology can play a key role by easing the access of pregnant women to the timely screening of GDM. One of the ways is through the development of an application where phone calls are made to the pregnant woman who is due for the screening of GDM. Currently, no system exists.

#### No system to follow-up deferred cases

In many instances, the OGTT is incomplete as the pregnant women experience vomiting and nausea. They are often asked to revisit the hospital to complete OGTT. However, according to HCPs, these women mostly do not revisit the hospital -either since they forget to turn up for screening or have other compelling reasons to miss screening.

An example of this challenge is demonstrated in the quote below:

*“In the first trimester, if women are complaining about any health issues, like vomiting, that time we cannot tell them to undergo GDM test. If we tell them to come again for the test, often they are left out, and that is the biggest challenge. We do not have such a system to call them for the test, like recording their contact numbers and calling them (over the phone to follow-up) to visit for the test.” (IDI#1, Obstetrician)*

##### Resource deficit

The Health system has to ensure adequate supplies that are required to conduct screening of GDM. However, it was found that one of the study sites was lacking glucose packets that are an important prerequisite for screening.

The obstetrician further adds the lack of basic supplies such as glucose packets needed for GDM screening as follows:

*“Despite being a government program, there is no supply of glucose packets; I think that should be made available. Then other things are not available here. I would like to have a counselor, in antenatal clinics. They will educate them about medical problems … … .” (IDI#3, Obstetrician)*

The obstetrician further stressed the need to have a counselor who could disseminate GDM related information to these pregnant women. To make matters worse, there is inequity at the macro health system level and unethical practices that go unmonitored as detailed below.

#### Inequity and unmonitored health system

According to a nurse, pregnant women who are not from urban areas have limited avenues for meeting the obstetrician and they end up consulting the wrong HCP. She highlights the unmonitored medical pluralism that exists in the study location and is largely true for India which poses hassle in directing these women to the right HCPs:

*“Women do not visit the hospital at the right time and they do not visit the right doctor. For example, in villages there will not be an obstetrician, so they would not have consulted any obstetrician. But they come here (city) for delivery. When you open their records, you find that they were consulting some Ayurvedic (Indian system of Medicine) obstetrician, sometimes pediatricians, and those with small shops. These women would not have received proper care and treatment or information. So, they will not be identified (screened) early. Educated women go for a check-up, but sometimes they might not receive proper guidance. Women in the working-class (laborers) often go to those obstetricians who may not be an obstetrician. So, I think people need the correct information.” (IDI#4, Nurse).*

**Fig. 2:**
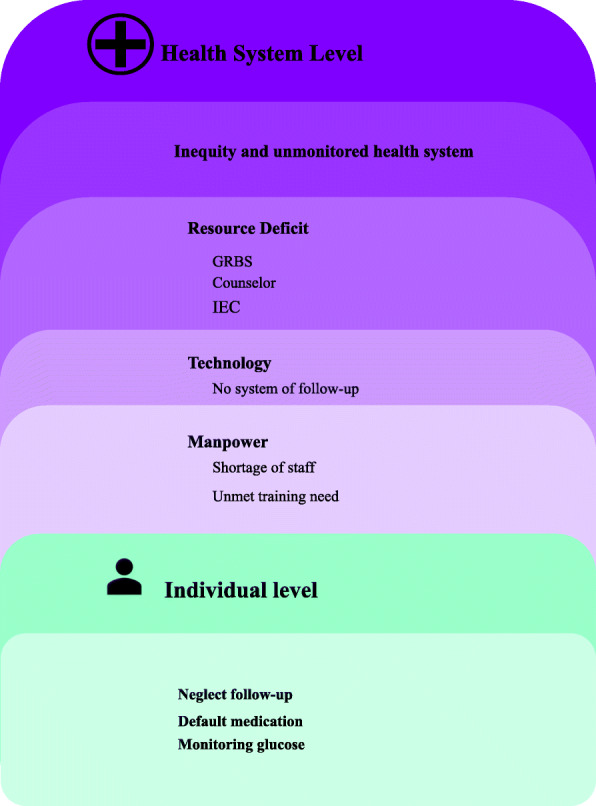
Socio-ecological model explaining the barriers to management of GDM in public hospitals of Bengaluru

When access to health is not based on the principle of equity and fair play, it is difficult to ensure that each pregnant woman can enjoy health during gestation by screening GDM on time and effectively manage it. In the given circumstances even an educated woman’s chance to meet the right HCP who guides for the timely screening of GDM might get compromised. After addressing the HCPs’ perspective of barriers that delay screening of GDM, next we will explore the HCPs’ perspective on barriers to the effective management of GDM (Fig. 2).

### Barriers to the effective management of GDM

The findings of our study that explores the barriers to the effective management of GDM from an HCP perspective is organized around different levels of the socio-ecological model, namely, at individual and health system levels. At the individual level, the women diagnosed with GDM are less likely to manage their condition effectively because they miss the follow-up visits to the health facility. We are already aware that women have many household responsibilities and tend to neglect their wellbeing and that could be a possible reason for them neglecting follow-up visits to the health facility. They also seem to skip GDM medication which the HCP assigns to women underestimating the risk that GDM poses for their wellbeing. Also, the reason for this underestimation could be due to counseling by HCPs that GDM is a condition that will go away after delivery. Also, monitoring sugar at home is especially difficult for women with GDM due to financial reasons or operational challenges. At the health system level barriers, lack of adequate manpower inhibits the transmission of information regarding the management of GDM. Also, the nurses are educating those diagnosed with GDM when they have not received any training to play that role. This can result in incomplete information being shared regarding the management of GDM, which could have bridged the knowledge gap and ensured handholding women for management of GDM. The resource deficit ranging from the lack of IEC material, glucose monitoring devices, strips, etc. makes the HCPs helpless for assisting those diagnosed with GDM to manage their condition more effectively. Finally, there is a lack of standard protocol for managing GDM, which creates utter chaos at the top level. That confusion is certainly permeating to other levels by finally hampering the health system’s readiness to manage GDM effectively. A more detailed thematic elaboration of the socio-ecological model that details the barrier to optimal management of GDM from the HCPs’ perspective that has emerged from the analysis is presented in the following section.

### Individual-level

The HCPs did not have a detailed understanding of the individual-level barriers that inhibited women diagnosed with GDM to manage their condition effectively. This lack of understanding might be due to the excessive workload that HCPs have as evident from the overcrowding at the health facility. Hence, HCPs have little time to engage deeply with women diagnosed with GDM and exploring why they have not been able to follow advice directed towards effectively managing GDM. However, we have a fair idea as to why women do not visit health facilities on time while exploring the reason for the delayed screening of GDM. A similar pattern is observed in the management of GDM as they default follow-up visits even after knowing their GDM status.

### Neglect of follow-up visit

Women who have been diagnosed with GDM miss follow-up visits to the health facility. A nurse participant honestly reveals that she does not know what personal problem compels women to miss these visits in her own words below:

*“Many a time women do not come for follow-up visits, I don’t know what problem they may have at home but they do not come for regular follow-up, suddenly after 2 months they come.” (IDI#4, Nurse).*

This reflection again highlights that our understanding of the barriers to the effective management of GDM can be complete only when we have explored the context at both the level of woman as well as HCPs.

### Neglect in intake of GDM medication

When women diagnosed with GDM do not take medication as prescribed by HCPs, it can deter optimum glycaemic control; hence poor management of GDM. A nurse participant observes that women with GDM tend to neglect the intake of medication as prescribed by the HCP.

*Even during pregnancy women do not take medicine correctly. Because we tell them that pregnancy diabetes (GDM) would go off after delivery, that may be a reason why they neglect tablets (medication). (IDI#4, Nurse).*

However, this participant is quick to reflect the reason behind such neglect. She believes that because HCP is counseling pregnant women with GDM that GDM is a condition that will resolve after delivery. Since the information provided is not accurate or complete, women underestimate risk and might be missing medication.

### Monitoring glucose

Again, monitoring sugar levels at the individual level do not come easily for everybody. Firstly, these women who have been diagnosed with GDM could be facing financial challenges in purchasing a glucometer or they could be struggling to use it. For instance, the obstetrician below says:

*“R: Yes, in-home they have to monitor glucose levels its is very difficult for them.*

*I: do they buy a glucometer?*

*R: Yes, some may buy it and for some, it is difficult (financial constraint). If they are on tablets we do not advise them to monitor. FBS and PPBS are checked here (at the health facility) only. Only for insulin patients, it needs to be monitored.” (IDI#1, Obstetrician)*

This indicates how the barrier to the monitoring of glucose could be a potential challenge for the effective management of GDM.

### Health system level

The barriers to proper management of GDM can be sub-categorized within the health system as health manpower, resource deficit, technology, and health system process.

### Manpower

As already indicated, the inadequacy of manpower in the health system makes it challenging for HCPs to provide the necessary support to pregnant women diagnosed with this condition and effectively manage it. The manpower challenge in the management of GDM is categorized as the shortage of manpower and task shifting without training.

### Shortage of manpower

Overcrowding at the public health facilities is very common in our study setting. These HCPs are overburdened with excessive workload and this is likely to deter optimal dispensing of information that is crucial for effective management of GDM.

#### Incomplete information on GDM being disseminated due to excessive workload

HCPs discussed the dissemination of GDM-related information based on the information that obstetricians and nurses provided to pregnant women with GDM. HCPs felt that information was often limited and only shared on a need-to-know basis. Women are advised by HCPs to undergo screening of GDM depending on the trimester of their visit to the hospital. However, they are not informed regarding the reason or relevance of the test.

Few HCPs said they would advise women with GDM to change their lifestyles, such as diet and exercise and they rarely explained the consequences of uncontrolled sugar levels for their unborn child. HCPs said that lack of time was the main reason for sharing limited information regarding GDM.

For instance, a nurse explains that women with GDM are often not informed of their condition by the obstetrician because they are overburdened with the workload, therefore in such situations, the nurses take the initiative to give a detailed explanation to such patients.

*“Sometimes, we (nurses) talk about it. Obstetricians also explain, but they would not have time, so we do it many times. Some (women) do not understand, so, on their slip, we write GDM, such that each time we see that we repeat (messages about diet control)” (IDI#2, Nurse)*

The obstetricians elaborated that if they have the time they provide detailed counseling for women and family members with high sugar levels. However, the borderline cases are not counseled for GDM screening as they do not have time:

*“We cannot explain it to every individual patient. To women with high levels, we may explain, but those who are borderline sugar level also require some diet tips or lifestyle changes tips. It is good if it (such information) is displayed to family members and her” – (IDI#3, Obstetrician)*

A nurse attributed their inability to give detailed information to pregnant women due to excessive workload as follows:

*“Obstetricians prescribe treatment. The same things we explain to the woman, that is all we can do when we sit in OPD. We cannot explain or follow-up in detail. Because there will be too much work and we do not have any assistance, so we could only provide detailed information or follow-up closely to 50% of women. Many women come to this hospital. We will not have time but to record their BP, check their height and weight, and document all this information into registers. It is a lot of work”.* (IDI#4, Nurse)

The shortage of manpower leading to excessive workload has emerged as the most important barrier which is inhibiting the sharing of information on the relevance of screening, the importance of GDM management, and its modalities.

### Nurses lack training in GDM management

As indicated by the nurse participants above, they often pitch in to explain to pregnant women diagnosed with GDM the dos and don’ts of GDM management. However, we have to keep in mind that these nurses have not received any systematic training for undertaking this role. Though they are doing their best to dispense GDM related information; however, due to lack of training, there is a greater likelihood that they might misinform these women. For instance, a participant reflects that because of their counseling where they say that GDM gets resolved after pregnancy could be a reason for women to underestimate the risk of GDM for their health and their unborn child and they neglect GDM medication.

### Technology

Technology has great potential in ensuring that women diagnosed with GDM can effectively manage their condition. The technology could automate reminders to be sent regarding the visit, test reports to be carried for consultation, lifestyle management information, etc. However, currently, no such system exists thus making timely screening and optimal management of GDM challenging.

#### No system to follow-up management of GDM

The HCPs explained that women who visited these public health facilities during the early stages of pregnancy were most likely to be advised by the Obstetrician and Gynaecologist (O&G) to undergo GDM screening. A nurse elaborated that only when these women are admitted, can they monitor GDM and its management. She lamented that pregnant women tend to forget what is prescribed:

*“I: How do you monitor sugar levels?*

*R: For those admitted to the hospital we monitor 3 times: morning, afternoon, and in the evening, we have to do that. ( … … .) Post-delivery, women stay for a week in the hospital, in that case, we monitor whether they take medicine or not. We can only do that. After they are discharged from the hospital, we do not know whether they follow whatever has been prescribed or told”. (IDI #5, Nurse)*

Currently, getting screened for GDM is a challenge and proper management of GDM seems even more elusive because HCP are not able to follow up on effective management of GDM. However, there is no mechanism to follow-up on further monitoring of the sugar levels in the postpartum phase. The same participant elaborates:

*“I: Do you have a system to follow -up women with GDM?*

*R: No, we do not have any system. Only when they come to the hospital, we ask them about their condition, that’s it. After delivery, we give enough tablets, but we do not know whether they take them or not. Some women do not even come for blood test post-delivery.” (IDI# 5, Nurse)*

Even during the early stages of being diagnosed with GDM, these women can be handheld with automated calls that remind them regarding their status and the steps to be undertaken for its effective management.

#### Resource deficit

The interviews with HCPs revealed that both the included hospitals are differently equipped to manage GDM cases. The general hospital at the tertiary level is better equipped to manage high-risk pregnancies compared to the referral center, a Community Health Centre (CHC). A nurse explained the lack of obstetrician in the night and other facilities to handle complicated cases of GDM as follows:

*“There was no such severe case. If we get any such case, we refer them to other hospitals, because this is a small hospital, and we do not have an obstetrician at night. We do not have facilities to manage complications, so we refer to other hospitals. If it is high (high sugar level), we refer to higher centers. We conduct only those deliveries which we can manage.” (*IDI#2, Nurse)

The CHC is equipped only to manage borderline GDM cases and refer cases to tertiary care for management and delivery. The HCPs at the CHC reported only the lack of Information, Education, and Communication (IEC) material related to GDM. IEC material is extremely crucial to inform women regarding the importance of effective management of GDM and if that is not available then how can these women expected to be screened on time and if diagnosed then effectively manage their condition.

However, the providers at the tertiary hospital reported a lack of glucose, only availability of damaged glucose monitoring machine, and lack of IEC materials (i.e., diet chart with necessary information) to hand out to women and their family members. Lifestyle changes through the change in diet and exercise cannot be stressed enough when it comes to the management of GDM. However, the study sites have reported a lack of a diet chart; thus, the health system has failed to provide the basics in informing women diagnosed with GDM for effectively managing their condition.

An obstetrician complained regarding the lack of infrastructure in hospitals essential to handle GDM cases and emphasized the need to inform policymakers regarding it as follows:

*“See just think how many General Random Blood Sugar (GRBS) machines working now (implied that GRBS machines are not working) if you admit them (women) if it (GRBS) is not working, then you are not able to monitor. Such things should not happen. GRBS machine is there but strips are not there. These are some technical problems we usually face here. Policymakers should be aware of all these and make some strict policy.” (*IDI# 3, Obstetrician)

The lack of these essential infrastructures at the hospital facility that is essential for effective management of GDM such as the GRBS machine and strips can severally impair the readiness of the health facility to effectively manage GDM during admissions.

#### Lack of standardized protocol for disseminating information on GDM management

The messages provided to women regarding GDM by the HCPs are less comprehensive**.** These messages are generic (do’s and don’ts) or cautionary - generating fear (e.g., if you do not control your sugar, it will affect your baby) as expressed by the obstetrician below:

*“She (woman with GDM) needs treatment, and she has to come for follow-up. If she does not come (for a check-up), it may affect fetal health, if she knows this much it is enough. We have no time to explain all things” (IDI#1, Obstetrician)*

A nurse also mentioned using *fear* as a strategy to ensure compliance on the part of women to take medication as follows:

*“I ask which month (of pregnancy) are you in, then see what the obstetrician has prescribed. And I explain which medicine to take before food and which one to take after food. I also tell them that if the medicine is not taken correctly, the baby would have some problems. If we tell them like this, she will take the medicine, because women care for baby more than herself. So, if I tell her that medicine is good for her (health), she may even neglect, so we talk (emphasize) about the baby.” (*IDI #4, Nurse.)

In the absence of disseminating standardized information and protocol for screening and management of GDM, the reality is that the messages received by pregnant women are not educative, but mostly instructive pieces of advice.

## Discussion

The public hospitals of Bengaluru city lack dedicated services for tackling GDM, and therefore many pregnant women have unmet health needs. Service providers recognize and support the need for the design and implementation of GDMSM services. Policymakers should consider adding new GDM services at public hospitals. While the obstetricians reportedly are aware of the national guidelines of GDM screening and its management, nurses have poor knowledge and by their admission would benefit from additional training. The health care providers are concerned regarding the poor implementation of GDM screening and management. In India, the guidelines followed for the management of GDM is not consistent with the WHO and FIGO guidelines [25]. While in India, diagnosis of GDM is done using OGTT, WHO guidelines recommend fasting plasma glucose and FIGO guidelines recommend HBA1c. Though Medical Nutrition Therapy is common in all the three guidelines, further pharmacological management is not common in these guidelines [24, 26, 28]. In other studies that were conducted in India to examine the diagnosis of management of GDM, it was found out that there was no consistency in the interventions being practiced. In a study conducted in Kerala, India, clinicians did not even uniformly adhere to the National Health Mission’s recommendation of the management of GDM. This was found even with clinicians treating patients in a single hospital [17, 18].

We identified several barriers in effectively managing the GDM in the women screened and diagnosed with GDM. The information provided to the pregnant women was often insufficient, and the HCPs did not convey the importance of timely and adequate management. This is complicated by the fact that standardized protocols for GDM management are not enforced in public hospitals. The lack of a qualified workforce at the public hospitals imposes an excessive workload on the HCPs. As a result, they do not get sufficient time to interact with pregnant women and explain GDMSM. We found that operational issues such as insufficient supplies and lack of equipment and reagents negatively impact GDMSM services. Also, there are missed opportunities for disseminating educational materials about GDMSM services to pregnant women, many of whom are literate and could benefit from written resources being made available.

With the view of enabling continuous service improvement, it is essential to initiate GDMSM services and set up monitoring and review mechanisms in public hospitals. Information regarding GDM and its complications to women need to be provided in the earlier phase of pregnancy. This step can improve the identification and treatment of pregnant women with GDM. HCPs rarely counsel patients with GDM on lifestyle modification, use of pharmacological therapy, or complications. There is limited evidence on the proportion of women with GDM who are diagnosed and managed with either drugs or lifestyle interventions. In high-income countries, there is a greater provision of individualized treatment goals and plans, and patients often have informed choices [29]. Ideally, HCPs give the information directly to the women with GDM, on the importance of maintaining normal blood glucose levels [30]. However, given the heavy workload of the HCPs in public hospitals, displaying infographics or videos to educate the women can be a valid alternative in improving self-care [15].

There are some communication barriers between HCPs and pregnant women. Communication training to HCPs and better documentation about the GDMSM and glucose values on the antenatal card can improve this. HCPs also reported the need for patient counselors, who can provide detailed information to them about GDM management.

Some HCPs reported that the attitude of the patient is a significant factor in determining whether the patients adhere to the treatment or not. However, evidence suggests that adequate knowledge of GDM and the treatment is a significant factor contributing to the compliance of the treatment [31]. HCPs opined that structured tools such as videos containing a step-by-step description of the disease and its management for educating the patients could improve treatment adherence. The role of an HCP is most significant for patients, mostly the illiterate and disempowered, who do not have access to other sources of authentic information. The cultural aspects of the patients are also essential, and healthcare providers need to adopt an inclusive style of providing care despite heavy workload [30].

The study had some limitations. Although a representative and relevant sample of service providers was identified, the sample size of our study was small. Also, the views of pregnant women will be essential when developing any new service. The responses of the service providers are specific to urban Bengaluru, which limits the generalizability to diverse areas in India and elsewhere. Health system challenges due to huge workload and lack of staff make it difficult for HCPs to implement standard guidelines. However, assessing the requirement of additional staff for implementing the GDM screening is beyond the scope of this study. The findings of this study organized around the socio-ecological model highlight how important it is to situate barriers to the timely screening of GDM and its effective management around the individual, household, cultural and health system context. Analyzing the problem uni-dimensionally cannot help us in gaining a deeper understanding of the problem at hand. This understanding of multiple levels is necessary for understanding and prioritizing the actionable points for implementing a program point. For instance, addressing the shortage of manpower, getting nurses trained in GDM, overcoming the resource deficit, and developing a standardized protocol of GDM screening and management can be necessary actions needed from the health system perspective. Also, larger issues of ensuring women have equal access to GDM screening and management irrespective of educational background and place of residence is a multi-sectoral issue and has to be addressed in that fashion.

## Conclusions

There is a need to develop GDMSM services to tackle the growing burden of GDM in pregnancy in India. There is sufficient evidence regarding the effectiveness of GDMSM services. These services need to be extended to public hospitals, and therefore the design of any new program requires consultation with service providers and service users.

## Supplementary Information

**Additional file 1.** In-depth Interview (IDI) Guide: Health Care Provider- Doctor.

**Additional file 2.** In-depth Interview (IDI) Guide: Health Care Provider- NURSE.

## Data Availability

The datasets generated and analyzed during the current study are not publicly available due to privacy issues but are available from the corresponding author on reasonable request.

## Abbreviations

GDM: Gestational Diabetes Mellitus
GDMSM: Gestational Diabetes Mellitus Screening and Management
IADSPG: International Association of the Diabetes and Pregnancy Study Groups
T2DM: Type 2 diabetes mellitus
OGTT: Oral Glucose Tolerance Test
CHC: Community Health Centre
MAASTHI: Maternal Antecedents of Adiposity and Studying the Transgenerational role of Hyperglycemia and Insulin
NICU: Neonatal Intensive Care Unit
HCPs: Health care providers
JGH: Jayanagar General Hospital
SRRH: Srirampura Referral Hospital
GCT: Glucose Challenge Test
GTT: Glucose Tolerance Test
FBS: Fasting Blood Sugar
PPBS: Postprandial Bloos Sugar
WHO: World Health Organisation
ANC: Antenatal Care
OPD: Outpatient Department
IEC: Information, Education and Communication; Institutional Ethics Committee
GRBS: General Random Blood Sugar
O & G: Obstetrician and Gynaecologist
