# Supplementary material for: Health care professionals’ perspectives on screening and management of gestational diabetes mellitus in public hospitals of South India – a qualitative study
Source: BMC Health Serv Res. 2021 Feb 12;21:133. doi: 10.1186/s12913-021-06077-0 (PMC7881531; doi:10.1186/s12913-021-06077-0)
Supplement: Supplementary file 2 — Additional file 2. In-depth Interview (IDI) Guide: Health Care Provider- NURSE. [file 12913_2021_6077_MOESM2_ESM.docx]

**INTERVIEW INFORMATION:**

| Interviewer code |  |
| --- | --- |
| Interview venue |  |
| Interview date |  |
| Interview start time |  |
| Interview end time |  |
| Total recorded time (hh/mm/) |  |

**RESPONDENT INFORMATION:**

| Respondent type | Nurse |
| --- | --- |
| Hospital Code |  |
| Designation |  |
| Gender |  |
| Age |  |
| Education/qualification |  |
| Experience (yy/mm) |  |

**OBSERVATION:**

- Look for IEC displayed in the waiting area (near the antenatal clinic).
- Look if there is anything specific on GDM (note/take pictures with permission from the hospital)

**Socio-ecological context**

**Individual context: client**

1. What is the profile (age, socio-economic and educational, language) of women who visit this hospital for pregnancy care?

2. Please tell us about the screening pregnant women:

1. What are some of the screening tests done?
2. When (at what month of pregnancy) are they done? Explain the process

***[if GDM is mentioned, first obtain the details of all the other screening tests]***

3. Now, we would like to know specifically about GDM screening.

1. In your hospital, are pregnant women screened for GDM? [ if no, ask Qn 16}
2. How common is GDM among women who visit your hospital?
   1. How many out of every 10 cases do you find women with GDM?
3. What type of women are more susceptible to GDM (age, ethnicity, region/place, education, socio-economic status)?

4. How important is it to screen women for GDM? Why?

1. How does it help women and her family?

5. Please explain the screening process- How does it start?

1. Who: Who (hospital staff) are involved in screening?
2. When: Is there any fixed day/ time for GDM screening? Why?
3. How: how many tests do women need to undergo to know her GDM status?
4. How do you prepare women for GDM screening?
   - 1. How do you plan screening activities?
     2. What are the conditions (if any) for the women to undergo GDM screening?
5. How much do you charge a woman to screen for GDM?

6. What happens after screening?

1. How many days does it take for women to get the test results?
2. Who discloses the GDM result to women?
   1. What is told to women with GDM?
   2. What is told to women with no GDM?

**Individual context: provider**

***Processes and role in GDM screening:***

7. What specific role do you play in screening?

**a. During screening/Diagnosis**: what do you do?

**b. Post-screening:** what do you do (e.g. GDM education/ messaging/ empathizing etc)?

1. Do you provide any information/messages to women about GDM?
2. Why/why not?
3. What messages do you provide?

8. How does screening women for GDM help a nurse?

9. What challenges do you face in screening women for GDM?

10. What are the feelings/emotions/concerns of women and her family expressed after learning the GDM status?

11. What treatment women with GDM receive?

12. Who guide women about the treatment procedures/ management of GDM?

13. What role do you play in the GDM management (check sugar levels)?

14. What challenges do you face in supporting women manage GDM?

15. What challenges do women face in the management of GDM? How does that affect you?

**Health system context**

***GDM Knowledge: Guidelines, Prevention, Treatment, consequences***

16. What are the National guidelines for screening women for GDM?

17. Please tell me about GDM. What do you know about GDM?

1. What causes GDM?
2. How to prevent it?

18. What are the available treatment options for those diagnosed with GDM?

1. Treatment types/criteria
2. Medications
3. Monitoring (of glucose)

19. When (at what stage of pregnancy) should women be diagnosed for GDM?

1. What happens GDM diagnosis is delayed?
2. What are the reasons/possibilities for delay?

20. How does GDM affect women?

1. During pregnancy
2. At the time of delivery
3. Post-delivery
4. How does it affect the baby?

**Health system training and resource**

21. Did you receive any training related to GDM?

1. What was the name of the training programme?
2. What was the content of the training programme (e.g. guidelines, management of GDM etc.)?
3. When did that happen? And, where?
4. How many days were you trained? How was it in terms of learning about GDM

22. Would you require further training? Why? Why not?

23. What support (materials/otherwise) would you require for the effective management of GDM?

24. What are some of the IEC material that you use/give away during antenatal check-up? (collect/ see the material/ take picture)

25. Do you have any IEC materials specific to GDM? (collect if any)

1. Would you require? Why/why not
2. According to you what kind of IEC materials (posters, flyers or video/ film etc. would be helpful for pregnant women? Why?

***Conclude the session thanking the participant. Stay there for about 10 minutes to ensure that the participant is comfortable, and get back to their social world (work).***

***Thank you for your participation and co-operation. Have a good day!***
